# Good Work Done, and to Be Done

**Published:** 1886-10-30

**Authors:** 


### GOOD WORK DONE, AND TO BE DONE.

"You hear that boy laughing? You think he's all fun;
But the Angels laugh, too, at the good he has done."

**Good Deeds at Home.** THE last few weeks have been memorable in the annals of good deeds done for the sick and suffering. At home, the work of Sister Dora has been commemorated by the unveiling of a statue at Walsall. She was, however, but a type of hundreds of noble women who labour yet, and who have long laboured in the hospitals and amongst the inhabitants of the poorer districts of our large towns. They do their work cheerily and well, but the world knows little of their self-denying lives, and cares less for the trials of those whom such devoted women live to tend and serve. Yet each and all, like the boy in Lowell's lines, make the angels laugh, too, at the good they have done and still do every day. Theirs is a noble calling, for they spend their lives, or much of their lives, in fulfilling the divine command, "Comfort ye, comfort ye My people." Who would not contribute to such work as this? Money all can give, and some can devote time and service, so all should co-operate in some way with the labourers, who are all too few at present, whilst the suffering and the sick increase and multiply every day.

**Good Deeds Abroad.** But good deeds are not the sole prerogative of one sex, or one nation, or one creed. The members of all creeds and nations, and the majority of individuals, too, are moved at times to noble aspirations, and by an intense longing to do good to their fellows. Take the history of the Roman Catholic priest, Father Damien, for example, as recorded in this week's papers. In the Sandwich Islands group, one island, Molokai, has been set apart for the reception of the lepers, who exist there in large numbers. No healthy or uninfected person ever dwelt on this island of Molokai, which was entirely given up to a population of upwards of six hundred lepers, until this young priest, some twelve years ago, was moved to go and live there, with the sole desire of bringing consolation and comfort to the unfortunate lepers. His was a mission as appalling as it was noble. The leper suffers from a peculiarly loathsome and repulsive malady. He has for this reason been isolated and treated as an outcast from the earliest days. Leper houses in this country and on the continent were the earliest, and indeed at one period the only, form of hospital. In the island of Molokai the young priest found none but lepers, yet he has valiantly laboured under these appalling conditions for upwards of twelve years, and now, alas, he has contracted the disease of leprosy himself. Labouring on, single-handed and alone, during all these years, Father Damien has performed for the lepers the offices of priest, physician, instructor, and even of provider, gardener, and cook. His life may be realised from the statement that he has not unfrequently had to dig the graves with his own hands before consigning his charges to their last resting-place. Who can read the bare recital of such devotion to duty, such noble self-denial, and such whole-hearted abnegation of self, without emotion? Many will doubtless be seized with a desire to do something in the day of their strength for those around them who need kindly succour, and whose lives might be greatly softened till the time of their deliverance arrives. Who is there amongst us all who does not hope or desire to have something sublime in his or her life, and, however faintly, still to leave behind them footprints on the sands of time?

**Causes of Inaction and Failure.** It is, we believe, the fact that all aspire to better things. But the great and preventing difficulty of the many is the lack of knowledge of how and where to begin to do good deeds for others. On the other hand, there are very many good people who work out their own good deeds in such quiet retirement that not even their best friends have the smallest conception of what they do for others. There are many agencies which only nominally do good, for the sufficient reason that, with the best intentions, the managers do not grasp the bearings of the work they nominally undertake. As a natural result, the material which they try to mould into a God-like image too frequently becomes even baser and more corrupt than before it came within the influence of their work. All this is, no doubt, very sad, but it is inevitable in this imperfect world in which we live. Wise men and women will not, therefore, waste valuable time in vain regrets, but each will determine to do something practical himself, so far as time and strength permit.

Good Work that wants Doing.

If any of our readers have arrived at such a stage in life's journey, we desire to claim their co-operation. The good deeds here recorded, and which move us so deeply, are but beacons to guide us on our way. For, remember, "all may do what has by man been done." The field of sickness and suffering, misery and death, is ever open to the faithful who labour in the love which gives them life. In every parish, amongst the poorer members of every congregation, there is to be found at least one sad case of human suffering in man, woman, or child, from time to time, which could be helped materially, if the necessary succour, based on knowledge, could be brought to bear upon it at the right time, and in the best way. Sir Andrew Clark, M.D., suggested an organisation the other day which we desire to see established. The difficult cases to which we refer might all be provided for. The delay and disappointment in searching for tickets for this or that charity might be easily reduced to a minimum, and much unavoidable suffering could be prevented, if Sir Andrew's scheme were to be put in force in every parish throughout the country. If there are, as there surely must be amongst our readers, men or women with time and a real desire to do their utmost as ministering angels, in an attempt to do good deeds whilst they have the strength for their accomplishment, we should be glad to receive, in confidence, their names and addresses, and the name of the place of worship they habitually attend. The details of Sir Andrew Clark's proposals will be shortly published, and it is in connection with them that we hope to hear of many workers from all parts of the country.

